# The astrocytic transporter SLC7A10 (Asc-1) mediates glycinergic inhibition of spinal cord motor neurons

**DOI:** 10.1038/srep35592

**Published:** 2016-10-19

**Authors:** Jeffrey T. Ehmsen, Yong Liu, Yue Wang, Nikhil Paladugu, Anna E. Johnson, Jeffrey D. Rothstein, Sascha du Lac, Mark P. Mattson, Ahmet Höke

**Affiliations:** 1 Department of Neurology, Johns Hopkins School of Medicine, 855 N. Wolfe Street, Baltimore, Maryland, 21205, USA; 2 Solomon H. Snyder Department of Neuroscience, Johns Hopkins School of Medicine, 725 N. Wolfe Street, Baltimore, Maryland, 21205, USA; 3 Medical Scientist Training Program, Johns Hopkins School of Medicine, 1830 E. Monument Street, Baltimore, Maryland, 21205, USA; 4 Laboratory of Neurosciences, National Institute on Aging Intramural Research Program, 251 Bayview Boulevard, Baltimore, Maryland, 21224, USA; 5 Brain Science Institute, Johns Hopkins School of Medicine, 855 N. Wolfe Street, Baltimore, Maryland, 21205, USA

## Abstract

SLC7A10 (Asc-1) is a sodium-independent amino acid transporter known to facilitate transport of a number of amino acids including glycine, L-serine, L-alanine, and L-cysteine, as well as their D-enantiomers. It has been described as a neuronal transporter with a primary role related to modulation of excitatory glutamatergic neurotransmission. We find that SLC7A10 is substantially enriched in a subset of astrocytes of the caudal brain and spinal cord in a distribution corresponding with high densities of glycinergic inhibitory synapses. Accordingly, we find that spinal cord glycine levels are significantly reduced in *Slc7a10*-null mice and spontaneous glycinergic postsynaptic currents in motor neurons show substantially diminished amplitudes, demonstrating an essential role for SLC7A10 in glycinergic inhibitory function in the central nervous system. These observations establish the etiology of sustained myoclonus (sudden involuntary muscle movements) and early postnatal lethality characteristic of *Slc7a10*-null mice, and implicate SLC7A10 as a candidate gene and auto-antibody target in human hyperekplexia and stiff person syndrome, respectively.

Motor neuron activity is tonically regulated by inhibitory currents, mediated through spontaneous and evoked synaptic release of glycine and GABA. Binding of glycine to postsynaptic glycine receptors results in an influx of chloride anions, hyperpolarizing the target cell and increasing the threshold for action potential generation.

Synaptic concentrations of glycine are modulated by two sodium-dependent transporters, SLC6A9 (GLYT1) and SLC6A5 (GLYT2). GLYT1 is primarily expressed by astrocytes but is also found within pre- and post-synaptic terminals of glutamatergic synapses throughout the central nervous system (CNS), where it likely plays a role in modulating both excitatory and inhibitory synaptic activity. GLYT2 is solely expressed in presynaptic terminals of glycinergic inhibitory neurons. Genetic mouse models for both of these transporters have been valuable for understanding their synaptic functions. Complete GLYT1 deficiency is associated with hypotonia and respiratory arrest due to altered pattern generation in brainstem respiratory centers due to excessive, tonic inhibition; thus the presumptive normal function of GLYT1 is clearance of glycine from inhibitory synapses and termination of glycinergic signaling^1^,^2^. GLYT2 deficiency, in contrast, is associated with hypertonia, ataxia, exaggerated startle responses called hyperekplexia, and early postnatal lethality; this is thought to reflect impaired reuptake of vesicular glycine into glycinergic neurons following glycine release (presynaptic recycling), leading to insufficient inhibitory activity^3^,^4^.

SLC7A10 (Asc-1) is a sodium-independent amino acid transporter known as the primary mediator of D-serine transport within the central nervous system^5^. Its primary function has been thought to be related to regulation of NMDA receptor activity at glutamatergic synapses via D-serine clearance^6^,^7^. Mice with homozygous deletion of *Slc7a10* display tremors, ataxia, rigidity, and seizure-like events that represent sustained myoclonus (sudden involuntary muscle movements)^6^; median lifespan is 21 days. This phenotype has been believed to reflect neuronal hyperexcitability arising from impaired transport of the NMDAR co-agonist D-serine^6^.

We observed that SLC7A10 is enriched in caudal regions of the brain, brainstem, and spinal cord. Knowing that SLC7A10 has high affinity and transport capacity for glycine^8^, and noting that its distribution correlates with regions of high-density glycinergic activity, we hypothesized that the phenotype of *Slc7a10*-null mice reflects impaired glycinergic inhibition. We show that SLC7A10 is substantially enriched in a subset of astrocytes, in contrast to previous reports of exclusive neuronal expression, and specify a primary role for this transporter in maintaining presynaptic neuronal glycine stores required for glycinergic inhibitory function.

## Results

### SLC7A10 expression corresponds to regions of high-density glycinergic inhibitory activity

**Figure 1:**
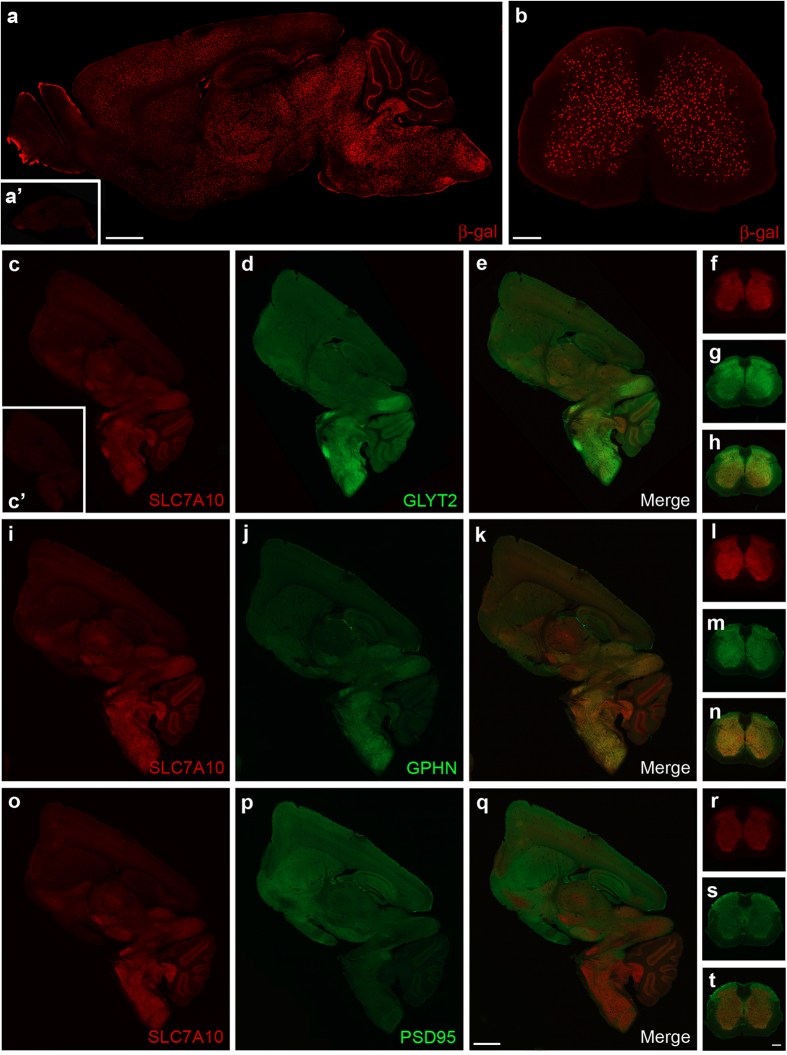
SLC7A10 expression is enriched in the caudal brain, brainstem, and spinal cord. Using beta-galactosidase (β-gal) as a surrogate marker for SLC7A10 expression, high-density expression is observed in caudal regions of the brain (**a**) and throughout the spinal cord grey matter (**b**), as well as in a select population of cells within the cerebellum (**a**). No beta-galactosidase expression is observed in knockout littermates lacking the targeted *Slc7a10* allele (i.e., wild type; a’). Regional distribution of endogenous SLC7A10 expression resembles areas of high density glycinergic inhibitory activity. Endogenous SLC7A10 distribution is similar to that of the presynaptic glycinergic marker GLYT2 in the brain (**c–e**) and spinal cord (**f–h**) and similar to that of the postsynaptic inhibitory marker gephyrin (GPHN) in both brain (**i–k**) and spinal cord (**l–n**). SLC7A10 expression appears complementary to post-synaptic density protein 95 (PSD95), a marker of excitatory glutamatergic synapses in brain (**o–q**) and spinal cord (**r–t**). *Slc7a10*^−/−^ brain shows complete absence of SLC7A10 immunostaining (c’). Scale bars, 1 mm (brain) and 500 μm (spinal cord).

Using an antibody detecting beta-galactosidase as a surrogate marker for SLC7A10 expression in a mouse line expressing beta-galactosidase directed by the endogenous *Slc7a10* promoter (*Slc7a10*^tm1Dgen^), we observed expression throughout the brain and spinal cord but noted a higher density of beta-galactosidase-expressing cells in the caudal brain, brainstem, and throughout the spinal cord grey matter. A discrete set of cells interspersed among cerebellar Purkinje cell bodies, likely Bergmann glia, also express beta-galactosidase (Fig. 1a,b).

To validate this expression distribution, we identified an antibody that specifically detects endogenous SLC7A10, and directly examined regional expression of SLC7A10 in brain and spinal cord sections along with markers characteristic of excitatory glutamatergic or inhibitory glycinergic and glycinergic/GABA-ergic synapses. Notably, SLC7A10 is enriched in regions where markers of glycinergic synapses show high expression (Fig. 1c–n). Specifically, SLC7A10 expression parallels the expression of GLYT2 (SLC6A5), a pre-synaptic glycine transporter highly enriched in glycinergic inhibitory neurons and considered a marker for these neurons (Fig. 1c–h), and gephyrin (GPHN), a post-synaptic glycine/GABA_A_ receptor clustering protein (Fig. 1i–n). Post-synaptic density protein-95 (PSD95), enriched at post-synaptic glutamatergic sites, shows complementary expression to SLC7A10, namely enriched in regions where SLC7A10 appears relatively low including throughout the cortex, hippocampus, and striatum (Fig. 1o–t).

### SLC7A10 is enriched in spinal cord and brainstem astrocytes

**Figure 2:**
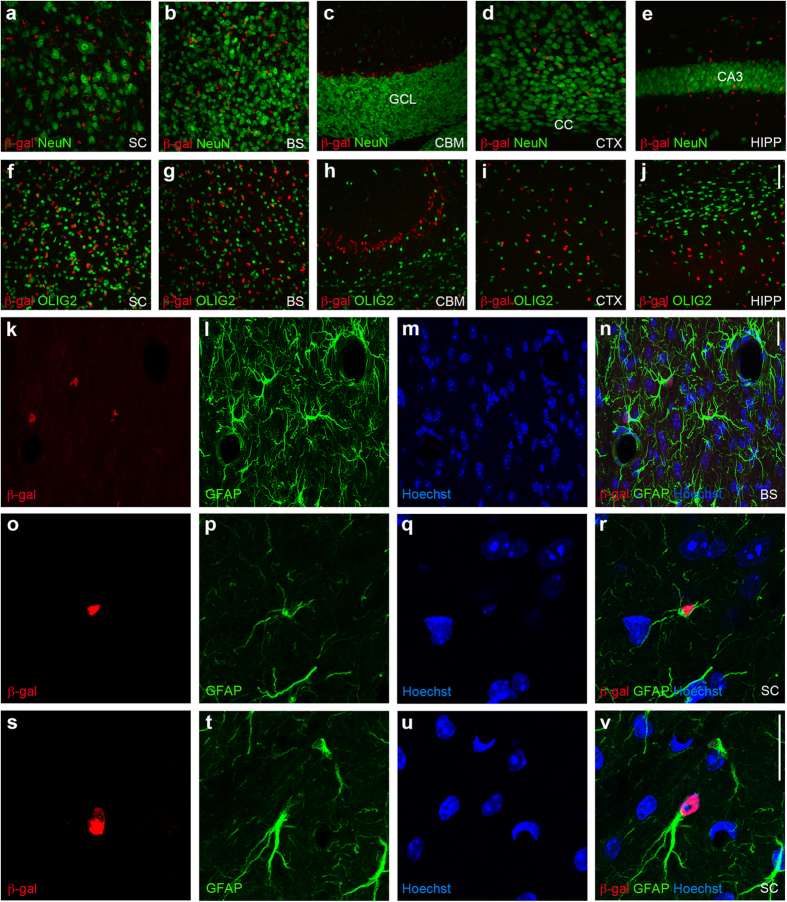
SLC7A10 is enriched in astrocytes. No beta-galactosidase expression (red) is co-localized with the neuronal nuclear marker NeuN (green) in the spinal cord (**a**, SC), brainstem (**b**, BS), cerebellum (**c**, CBM), cortex (d, CTX), or hippocampus (e, HIPP) of *Slc7a10*^+/−^ mice. GCL, granule cell layer; CC, corpus callosum. No beta-galactosidase expression (red) is co-localized with oligodendrocyte transcription factor 2 (OLIG2), an oligodendrocyte marker (green) in the spinal cord (**f**, SC), brainstem (**f**, BS), cerebellum (**f**, CBM), cortex (**i**, CTX), or hippocampus (**j**, HIPP). All beta-galactosidase-positive cells in the brainstem (k) appear to be astrocytes, as identified by staining for glial fibrillary acidic protein (GFAP) (**l**,**n**). Representative beta-galactosidase-positive cells in the spinal cord co-stained for GFAP are shown in o-b. Scale bars, 50 μm (**a**–**j**), 20 μm (**k–v**).

Earlier studies describe SLC7A10 as a neuronal transporter^9^,^10^,^11^. Unexpectedly, we do not detect co-localization of beta-galactosidase expression with the neuronal nuclear marker NeuN within the cortex, hippocampus, brainstem, cerebellum, or spinal cord of heterozygous *Slc7a10*^tm1Dgen^ mice (Fig. 2a–e). To delineate the identity of beta-galactosidase-expressing cells, we evaluated co-localization with the oligodendrocyte marker oligodendrocyte transcription factor 2 (OLIG2) and the astrocytic marker glial fibrillary acidic protein (GFAP). We do not observe co-localization of beta-galactosidase and OLIG2 within the cortex, hippocampus, brainstem, cerebellum, or spinal cord (Fig. 2f–j). However, beta-galactosidase-positive cells co-localize with GFAP (Fig. 2k–v). Notably, not all astrocytes appear to express beta-galactosidase, suggesting restricted expression of SLC7A10 to a subset of astrocytes; the proportion of astrocytes expressing beta-galactosidase appears to be higher in caudal brainstem and spinal cord regions, as noted earlier for endogenous SLC7A10 expression (Fig. 1c–t).

**Figure 3:**
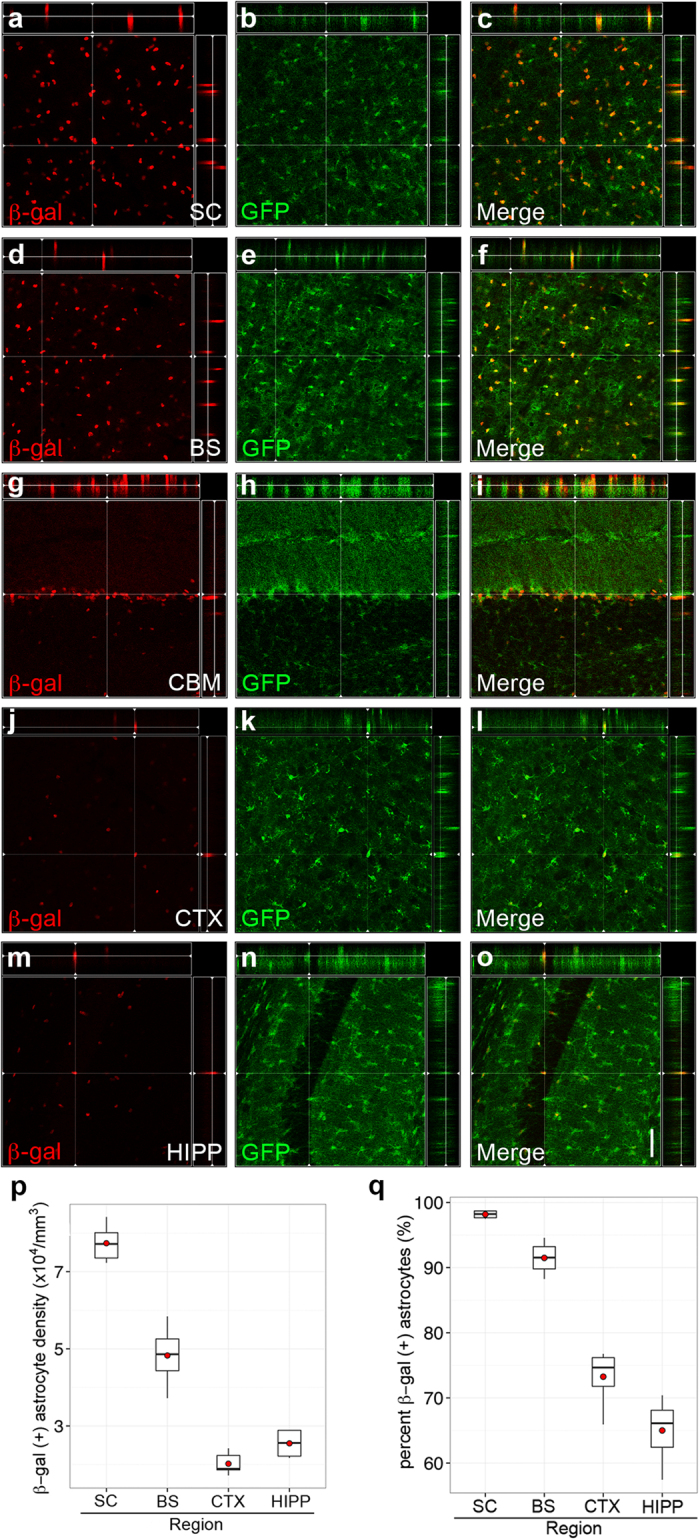
SLC7A10 is enriched in astrocytes in regions of high-density inhibitory activity. Mice hemizygous for *GLT1*-GFP and heterozygous for *Slc7a10* show beta-galactosidase expression exclusively in GFP-positive astrocytes in the spinal cord (**a–c**, SC), brainstem, (**d–f**, BS), cerebellum (**g–i**, CBM), cortex (**j–l**, CTX), and hippocampus (**m–o**, HIPP). Significantly higher densities of beta-galactosidase-positive astrocytes are observed in the spinal cord compared to all other brain regions examined (p < 0.001), and significantly higher densities are present in the brainstem compared to cortex and hippocampus (p < 0.001) (**p**). Similarly, the percentage of beta-galactosidase-expressing astrocytes is significantly higher in spinal cord and brainstem compared to cortex and hippocampus (p < 0.001) (**q**). Orthogonal views are shown for each region, demonstrating colocalization of GFP and beta-galactosidase signals. n = 3 biological replicates for each genotype. Scale bar, 50 μm.

Because antibody-based GFAP detection can show variability among different brain regions, we bred *Slc7a10* heterozygous mice with a BAC-transgenic mouse line expressing GFP under control of the ubiquitous astrocytic marker glial glutamate transporter 1 (SLC1A2, or GLT1). In all CNS regions examined (spinal cord, brainstem, cortex, hippocampus, cerebellum), beta-galactosidase-positive cells co-localize with GFP, validating astrocytic enrichment of SLC7A10 throughout the central nervous system (Fig. 3a–o).

We quantified the proportion of astrocytes expressing SLC7A10 in the brain regions indicated, and find a significantly higher density of beta-galactosidase-expressing astrocytes in the spinal cord and brainstem compared to all other CNS regions examined [Fig. 3p, (7.7 ± 0.2) × 10^4^/mm^3^, (4.8 ± 0.3) × 10^4^/mm^3^, (2.0 ± 0.1) × 10^4^/mm^3^, (2.5 ± 0.2) × 10^4^/mm^3^ for spinal cord ventral horn, brainstem, cortex, and hippocampus, respectively, mean ± SEM; p < 0.001 for spinal cord compared to all other brain regions]. The proportion of beta-galactosidase-positive astrocytes is also significantly higher in spinal cord compared to all other CNS regions examined (Fig. 3q, 98 ± 0.2%, 91.5 ± 1%, 73 ± 1.7%, 65 ± 2% for spinal cord ventral horn, brainstem, cortex, and hippocampus, respectively, mean ± SEM; p < 0.001 for spinal cord compared to cortex and hippocampus, p = 0.02 for spinal cord compared to brainstem). We did not detect any differences in cellular expression patterns among early postnatal (P2), young (P21), and adult (P56) mice.

**Figure 4:**
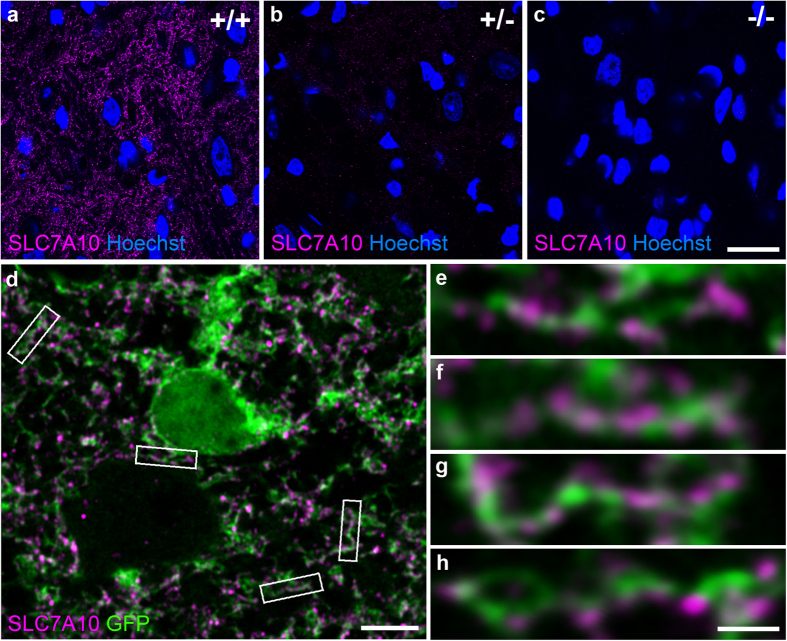
Endogenous SLC7A10 expression is consistent with astrocytic enrichment. An antibody that detects endogenous SLC7A10 shows specific graded expression in wild type, heterozygous, and knockout mice (**a**–**c**). Super-resolution imaging of brainstem sections from *GLT1*-GFP mice expressing cytoplasmic GFP in astrocytes shows astrocytic processes decorated with punctate SLC7A10 labeling (**d–h**). Scale bars, 20 μm (**a–c**), 5 μm (**d**), 1 μm (**e–h**).

To extend these reporter gene-based observations and confirm that endogenous SLC7A10 expression is also enriched in astrocytes, we used an antibody that specifically detects SLC7A10 to label endogenous SLC7A10 in brainstem sections from mice expressing cytoplasmic GFP directed by the GLT1 promoter (Fig. 4a–h). The antibody used specifically detects SLC7A10, as demonstrated by graded labeling in wild type and heterozygous brain sections, and absent labeling in knockout animals (Fig. 4a–c). Super-resolution imaging shows that SLC7A10 labeling co-localizes with astrocytes expressing cytoplasmic GFP (Fig. 4d–h). These findings are consistent with the beta-galactosidase reporter localizations described directly above; altogether, these data strongly support the conclusion that SLC7A10 is enriched in astrocytes.

### Total spinal cord glycine levels are reduced by SLC7A10 deficiency

**Figure 5:**
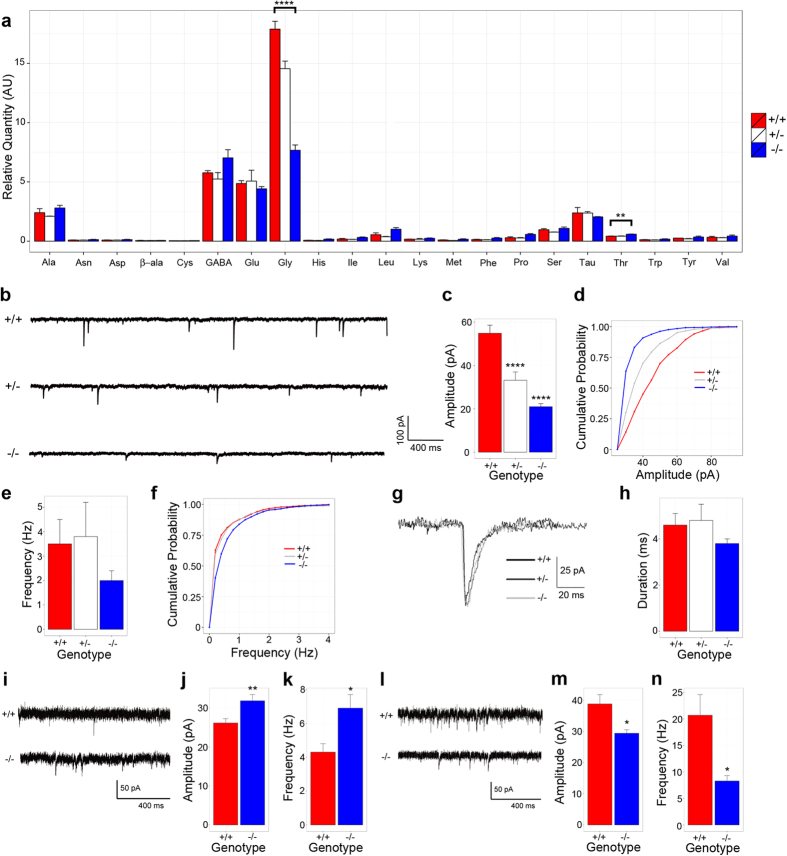
*Slc7a10* deletion is associated with decreased spinal cord glycine levels. Whole spinal cord lysates from P21 mice were assayed for amino acid content by ALEX-CIS GCTOF (**a**). Mean glycine levels are significantly decreased in *Slc7a10*-null mice compared to heterozygous and wild type littermates (****p < 0.0001). Mean threonine levels are significantly increased in *Slc7a10*-null mice compared to heterozygous and wild type littermates (**p = 0.003). No significant differences were detected among wild type and heterozygous littermates. **SLC7A10 deficiency is associated with reduced motor neuron glycinergic mIPSC amplitudes**. Glycinergic mIPSC traces from *Slc7a10* wild type, heterozygous, and knockout mice (**b**). Mean glycinergic mIPSC amplitudes are significantly reduced in heterozygous and knockout mice compared to wild type (****p < 0.0001) (**c**,**d**). Mean glycinergic mIPSC frequency does not significantly differ among genotypes (p = 1.0 for wild type vs. heterozygote, p = 0.4 for wild type vs. knockout) (**e**,**f**). Normalized average waveforms of glycinergic mIPSCs from spinal cord motor neurons (**g**). Mean decay times do not differ among *Slc7a10* genotypes (**h**). n ≥ 10 total cells from ≥ 3 biological replicates. **GABAergic mIPSC amplitude and frequency is significantly increased in** ***Slc7a10*****-null spinal cord motor neurons**. Mean GABA-mediated mIPSC amplitudes were significantly increased in *Slc7a10*-null motor neurons (**i**,**j**) (amplitude, in pA, 31.9 ± 1.6 vs. 26.2 ± 1.1, knockout vs. wild type, **p = 0.008). Mean GABA-mediated mIPSC frequencies were also significantly increased (**k**) (frequency, in Hz, 6.9 ± 0.8 vs. 4.3 ± 0.5, knockout vs. wild type, *p = 0.01). n = 11 total cells from 3 biological replicates. **NMDAR- and AMPAR-mediated glutamatergic transmission is significantly diminished in** ***Slc7a10*****-null spinal cord motor neurons**. Excitatory currents in *Slc7a10* wild type and knockout mice (**l**). Glutamatergic currents are diminished in *Slc7a10*-null mice compared to wild type (m, amplitude, in pA, 29.4 ± 1.1 vs. 38.8 ± 3.0, knockout vs. wild type, *p = 0.01; n, frequency, in Hz, 8.3 ± 1.0 vs. 20.7 ± 3.9, knockout vs. wild type, *p = 0.01). n = 11 total cells from 3 biological replicates. Error bars indicate mean ± SEM.

We elected to examine spinal cord content of amino acids known to be transported by SLC7A10 or known to have inhibitory capacity (Fig. 5a). With correction for multiple comparisons, only the difference in mean glycine and threonine levels among *Slc7a10*^+/+^ and *Slc7a10*^−/−^ mice achieved statistical significance. Glycine levels are substantially decreased in *Slc7a10*^−/−^ spinal cord compared to *Slc7a10*^+/+^ or *Slc7a10*^+/−^ [mean glycine levels (178.9 ± 6.6) × 10^4^ vs. (76.7 ± 4.5) × 10^4^ for wild type and knockout, respectively; p < 0.0001]. Threonine levels are modestly increased in *Slc7a10*^−/−^ spinal cord compared to *Slc7a10*^+/+^ or *Slc7a10*^+/−^ [mean threonine levels (41.9 ± 1.9) × 10^3^ vs. (58.4 ± 1.8) × 10^3^ for wild type and knockout, respectively; p = 0.003]. Of note, we did not detect significant differences among any genotypes in mean levels of other L-amino acids transported by SLC7A10, including serine, alanine, and cysteine, or other amino acids with inhibitory capacity including GABA, β-alanine, and taurine. We did not detect any significant differences among the amino acids assessed in the cerebellum (data not shown).

### *Slc7a10* deletion is associated with diminished glycinergic inhibitory activity

To directly test whether SLC7A10 deficiency impairs glycinergic inhibitory transmission, we isolated and recorded glycinergic miniature inhibitory post-synaptic currents (mIPSCs) from spinal cord slices of wild type, heterozygous, and *Slc7a10*-null littermates (P10-P13). Motor neurons from *Slc7a10* heterozygous and *Slc7a10*-null mice show significantly reduced glycinergic mIPSC amplitudes compared to wild type mice (Fig. 5b–d; in pA, 54.9 ± 3.7, 33.2 ± 3.8, and 21.0 ± 1.4 for wild type, heterozygous, and knockout, respectively; p = 3.1 × 10^−11^ for wild type vs. knockout and p = 2.8 × 10^−5^ for wild type vs. heterozygote). Glycinergic mIPSC frequencies of heterozygous and knockout mice do not differ significantly from wild type mice (Fig. 5e,f; p = 1.0 and p = 0.4, respectively). We did not detect any significant differences in glycinergic mIPSC duration among any genotypes (Fig. 5g,h).

To determine whether SLC7A10 deficiency specifically impairs glycinergic inhibitory transmission, or whether this could be a manifestation of more global defects related to disrupted neuronal activity, we evaluated GABAergic inhibitory transmission as well as NMDAR- and AMPAR-mediated glutamatergic transmission in *Slc7a10*-null mice compared to wild type littermates (Fig. 5i–n). Notably, the mean amplitude of GABAR-mediated currents is significantly increased in *Slc7a10*-null motor neurons (Fig. 5i,j; p = 0.008). The mean frequency of GABAR-mediated currents is also significantly increased (Fig. 5i,k; p = 0.01). Conversely, glutamatergic NMDAR- and AMPAR-mediated currents are significantly decreased in both frequency and amplitude in *Slc7a10*-null motor neurons (Fig. 5l–n; p = 0.01 for frequency, p = 0.01 for amplitude).

**Figure 6:**
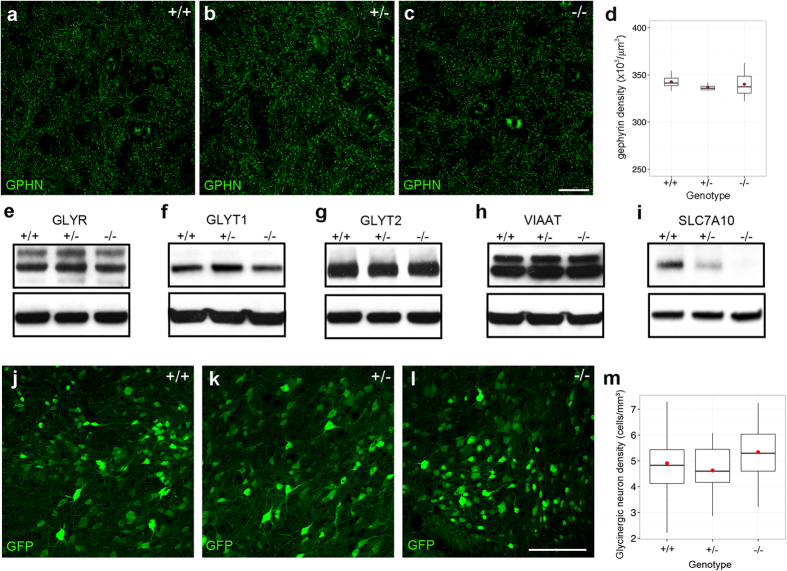
mIPSC alterations are independent of inhibitory synapse number. Total inhibitory synapse density does not differ among *Slc7a10* genotypes, as assessed by gephyrin (GPHN) density in the spinal cord ventral horn of P10-P12 mice (p = 1.0 for wild type vs. knockout; p = 1.0 for wild type vs. heterozygote; p = 0.9 for wild type vs. heterozygote) (**a**–**d**). n = 3 biological replicates for each genotype. **Glycinergic synaptic architecture is grossly unaltered among** ***Slc7a10*** **genotypes**. Total spinal cord content of the glycine receptor (GLYR, **e**), GLYT1 (**f**), GLYT2 (**g**), and VIAAT (**h**) does not differ among wild type, heterozygous, and knockout animals. Beta-tubulin loading controls are shown below each blot (the same loading control was used for GLYR and GLYT2). n = 3 biological replicates for each genotype; p > 0.3 for all pairwise comparisons except for SLC7A10 (p < 0.02 for differences among genotypes). Full-length immunoblots are presented in Supplementary Figure S1. **SLC7A10 deficiency does not cause loss of spinal cord glycinergic neurons**. Glycinergic neuron density does not differ among genotypes, as assessed by count of GFP-positive cells in spinal cords of P10-P12 mice, in which GFP expression is directed by the promoter of the glycinergic neuronal marker GLYT2 (**j**–**m**) (p = 0.7 for wild type vs. knockout, p = 1.0 for wild type vs. heterozygote; p = 0.1 for heterozygote vs. knockout). n = 3 biological replicates for each genotype. Scale bar, 100 μm.

To determine whether the reduced glycinergic mIPSC amplitudes we observed in *Slc7a10*-null mice could reflect alterations in total inhibitory synaptic number, we evaluated total inhibitory synapse density using the post-synaptic inhibitory marker gephyrin (GPHN) (Fig. 6a–d). We did not observe any differences among any genotypes (p = 1.0 for wild type vs. knockout, p = 0.9 for wild type vs. heterozygote).

We further assessed the possibility of altered inhibitory synaptic architecture by examining crude nervous system content of the glycine transporters GLYT1, GLYT2, vesicular inhibitory amino acid transporter (VIAAT), and total glycine receptor content (GLYR) (Fig. 6e–i). We did not observe differences among any genotypes for any of the proteins examined [p > 0.3 for all pairwise comparisons except for SLC7A10 (p < 0.02 for differences among genotypes)].

To exclude the possibility that absence of SLC7A10 could cause death or deficiency of glycinergic neurons, we bred *Slc7a10* heterozygous mice with mice expressing GFP directed by the glycinergic transporter 2 (GLYT2) promoter; as mentioned earlier, this is a specific marker for glycinergic inhibitory neurons (Fig. 6j–m). Spinal cord densities of glycinergic neurons (determined by counting GFP-positive neurons) do not differ among genotypes [(4.9 ± 0.3) × 10^4^/mm^3^, (4.6 ± 0.2) × 10^4^/mm^3^, (5.3 ± 0.2) × 10^4^/mm^3^ for wild type, heterozygous, and knockout mice, respectively; mean ± SEM; p = 1.0 for wild type compared to heterozygous, p = 0.7 for wild type compared to knockout, p = 0.1 for heterozygous compared to knockout]. Thus, SLC7A10 deficiency does not appear to influence survival of glycinergic inhibitory neurons.

## Discussion

Our findings demonstrate an essential role for SLC7A10 in maintaining glycinergic inhibitory function. Both GABA and glycine are loaded into presynaptic vesicles via the vesicular transporter VIAAT; this transporter, however, has a low affinity for glycine, which mandates specific mechanisms for enrichment of glycine within glycinergic neurons in order to sustain vesicular loading^12^. Glycine is enriched 10- to 100-fold in glycinergic neurons, yet these neurons do not have a known dedicated enzymatic mechanism for generating these high concentrations. Basal synthesis alone does not explain the enrichment of glycine in glycinergic inhibitory neurons, an observation that emphasizes the importance of a mechanism for local enrichment and/or recapture^13^.

*Slc6a5*-null (GLYT2-deficient) mouse mutants develop spasticity and die during the second postnatal week; in these animals glycinergic mIPSCs are significantly reduced in amplitude and frequency, which has been ascribed to a deficit in presynaptic recycling of glycine^3^. We find that the phenotype of *Slc7a10*-null mice essentially phenocopies that of *Slc6a5*-null mice, including features of rigidity and myoclonus characteristic of the clinical condition called hyperekplexia (literally meaning “exaggerated surprise”), along with electrophysiologic evidence of specific glycinergic dysfunction.

We observed a significant reduction in glycinergic mIPSC amplitudes in spinal cord motor neurons of *Slc7a10*-null mice, which we interpret as a demonstration of decreased vesicular quantal size (i.e., per-vesicle glycine content) within glycinergic interneurons. These findings are consistent with a model in which glycinergic inhibitory neurons are chronically depleted of glycine in the absence of a sustained supply of glycine or its precursors from astrocytes, via SLC7A10. Our observation that mice heterozygous for *Slc7a10* show mIPSC amplitudes intermediate to those of *Slc7a10*-null mice and wild type mice is consistent with a concentration-dependent model of quantal insufficiency. Our findings are distinct from an independent study that described a decrease in frequency, but not amplitude, of glycinergic mIPSCs in brainstem hypoglossal neurons of *Slc7a10*-null mice^14^. While we observed a non-significant trend towards a reduction in glycinergic mIPSC frequency in *Slc7a10*-null motor neurons, we suggest that this may be more related to a limitation in identifying individual events due to small amplitudes among noise, rather than a true decrease in frequency. Very similar glycinergic mIPSC features that we describe here for *Slc7a10*-null mice have been described for mice lacking the glycine transporter GLYT2, mutations in which are a known cause of hyperekplexia in humans; these parallels provide compelling support for the interpretation that the phenotype of *Slc7a10*-null mice represents hyperekplexia^3^,^15^.

Our observations that NMDAR/AMPAR-mediated glutamatergic excitatory transmission is diminished in motor neurons of *Slc7a10*-null mice, while GABAergic inhibitory transmission is significantly increased, are consistent with a model in which glycinergic neurotransmission is a primary deficit, with corresponding changes in glutamatergic and GABAergic transmission likely reflecting homeostatic compensation in the setting of chronic hyperexcitability. As such, *Slc7a10*-null and conditional mice may also represent a novel model for studying mechanisms of homeostatic plasticity.

In contrast with earlier studies, our findings show that SLC7A10 is enriched in astrocytes with especially high density in brainstem and spinal cord regions, correlating with higher densities of glycinergic inhibitory neurons in these areas. This discrepancy in cell identity may be due to quality of antibodies used in previous studies or difficulties in distinguishing fine astrocytic processes from neuropil. Cell type-specific conditional deletion of *Slc7a10* will definitively address these issues. Meanwhile, our immunolocalizations are supported by four independent transcriptional profiling studies that identify *Slc7a10* mRNA among a set of astrocyte-enriched transcripts^16^,^17^,^18^,^19^,^20^. We conclude that *Slc7a10*-null mice represent a novel disease model for hyperekplexia, and reveal an astrocytic origin for the phenotype in addition to presently known dysfunction of pre- and post-synaptic elements of glycinergic inhibitory neurons.

**Figure 7:**
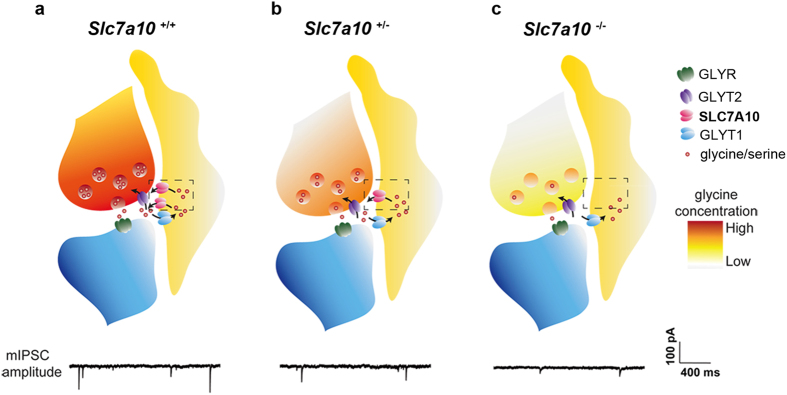
Astrocytic SLC7A10 mediates glycine enrichment in glycinergic inhibitory neurons. Either via direct supply of glycine, or through supply of biosynthetic precursors such as L-serine, astrocytic SLC7A10 mediates the cytoplasmic enrichment of glycine in glycinergic inhibitory neurons required to maintain adequate loading/quantal content of glycinergic synaptic vesicles. This model predicts that *Slc7a10*-null mice are unable to supply or recycle glycine (or glycine precursors) from astrocytes to glycinergic neurons; insufficient cytoplasmic concentration of glycine within glycinergic neurons leads to decreased vesicular glycine quanta, as reflected in diminished glycinergic mIPSC amplitudes and a hyperekplexia-like phenotype. Our observation that mice heterozygous for *Slc7a10* show mIPSC amplitudes intermediate to those of *Slc7a10*-null mice and wild type mice is consistent with a concentration-dependent model.

We propose that astrocytes play a role in supplying and/or recycling glycine or its precursors (i.e., L-serine) to glycinergic neurons via SLC7A10 (Fig. 7). Experiments assessing acute and chronic effects of SLC7A10 inhibitors on glycinergic transmission may help distinguish whether this function occurs on a time-scale associated with synaptic transmission, or whether SLC7A10 may be involved in basal supply of these substrates. The absence of a sodium requirement for transport (in contrast to the other known glycine transporters GLYT2 and GLYT1) is consistent with a role for SLC7A10 in glycine efflux. Basal astrocytic glycine levels appear to be low, which has been attributed to metabolism by the glycine cleavage system (GCS), an astrocyte-enriched multi-enzyme mitochondrial complex that metabolizes glycine to ammonia, carbon dioxide, and a methylene group^21^,^22^,^23^,^24^; however, rapid release could also partially explain these observations. The GCS is the primary route of glycine catabolism in mammals, and is known to have a high rate of activity. Isotopic glycine studies in humans show that the GCS produces methylene units at ~20 times the rate required for methylation demand; most of these one-carbon units are used to synthesize serine or become oxidized to carbon dioxide^22^,^25^.

Alternatively, astrocytic SLC7A10 may be required to enrich glycinergic interneurons with L-serine as a precursor for glycine synthesis. In the absence of SLC7A10, the glycine pool typically maintained within glycinergic interneurons may never be synthesized. However, mice with specific astrocytic deletion of 3-phosphoglycerate dehydrogenase (*Phgdh*), an enzyme required for astrocytic L-serine synthesis and L-serine provision to neurons, show normal survival and have not been reported to display phenotypes resembling glycinergic dysfunction, despite 60% reduction in cortical glycine levels and 80% reduction in L-serine^26^.

We observed that P10-P13 heterozygous *Slc7a10* mice also show significantly reduced glycinergic mIPSC amplitudes in spinal cord motor neurons compared to wild type mice, yet remain grossly asymptomatic and show normal survival. Whether this physiologic deficit persists into adulthood, and whether it confers any susceptibilities, remains to be explored. Recently described spontaneous GLYT2-heterozygous animals were found to have normal thermal sensitivity but enhanced repetitive grooming and hyperactivity in the elevated plus-maze test^27^; these behaviors were interpreted as representative of decreased glycinergic facilitation of NMDARs, i.e., NMDAR hypofunction^28^.

NMDA receptor hypofunction is thought to play a role in the symptoms of schizophrenia, and modulation of NMDA receptor activity is an actively explored pharmacologic target. In this regard inhibition of glycine transporters has already been shown to alleviate negative symptoms in schizophrenia^29^. GLYT1 blockade enhances NMDAR signaling^30^,^31^,^32^ and synthetic GLYT1 inhibitors are currently in clinical trials to enhance NMDAR function^29^. For similar reasons selective inhibitors of SLC7A10 are also being actively considered as therapeutic options for the treatment of schizophrenia^33^. Our findings have important implications for these approaches.

Defects in GABAergic and glycinergic inhibitory transmission among spinal cord dorsal horn circuits are known to contribute to neuropathic and inflammatory pain states^34^, and GLYT2 blockade is known to have an analgesic effect^29^, suggesting the possibility of a role for SLC7A10 in pain modulation as well. Augmentation of astrocytic SLC7A10 activity could theoretically represent an additional target toward therapeutic goals as an anti-convulsant, analgesic, or muscle relaxant. While GABA_A_ receptors are common targets for these purposes (e.g., benzodiazepines), modulation of glycinergic pathways are presently less developed.

Finally, as alluded to earlier, mutations in or autoantibodies against various components of glycinergic synaptic architecture yield phenotypes associated with hypertonia and exaggerated startle reflexes, called hyperekplexia and stiff person syndrome, respectively^35^,^36^. The majority of known human hyperekplexia mutations comprise missense and nonsense mutations in *GLRA1*, the gene encoding the GLYR α1 subunit and *SLC6A5*, the gene encoding the presynaptic GLYT2 transporter^15^,^37^,^38^. Mutations have been identified in genes encoding other glycinergic components as well, including the GLYR B subunit (*GLRB*)^39^, collybistin (*ARHGEF9*)^40^, and the post-synaptic GlyR/GABAR clustering protein gephyrin (*GPHN*)^41^,^42^. A large proportion (35%) of human families with clinically diagnosed hyperekplexia remain “gene-negative,” meaning that causative mutations have not been identified in any of the known hyperekplexia disease genes. Current efforts to expand the characterization of these conditions have logically focused on other components of the glycinergic apparatus, including VIAAT (the GABA/glycine vesicular transporter), ULIP6 (a GLYT2-interacting protein which may play a role in GLYT2 endocytosis and recycling), and syntenin-1 (a PDZ-containing protein that also interacts with GLYT2 and may be important for trafficking and/or pre-synaptic targeting of GLYT2)^35^,^43^,^44^. Our findings highlight the possibility that mutations in human *SLC7A10* or autoantibodies against human SLC7A10 could underlie a subset of the remaining gene-negative hyperekplexia families or auto-antibody negative individuals with stiff person syndrome.

## Methods

### Animal husbandry

*Slc7a10*^tm1Dgen^ mice were obtained from Deltagen (San Mateo, CA). *GLYT2*-eGFP and BAC *GLT1*-eGFP mice have been previously described^45^,^46^. Male and female mice were used equally, and where possible all studies were conducted on matched littermates. Mice were genotyped by quantitative PCR through Transnetyx (Cordova, TN). Experiments were performed in accordance with protocols approved by the Animal Care and Use Committee at Johns Hopkins University.

### Immunofluorescence

P2, P16-P21, or 8–10 week-old mice were deeply anesthetized with 0.4 mg/g 2,2,2-tribromoethanol (Avertin), then perfused transcardially with 4% paraformaldehyde in 0.1 M phosphate buffer, pH 7.4 (PB), post-fixed overnight at 4 °C, and cryoprotected in 30% glycerol in 0.1 M PB overnight. 12–40 μm tissue sections were cut on a freezing microtome and processed free-floating. Primary antibodies were diluted in PBS containing 5% goat serum and 0.3% triton-X-100, and incubated with tissue sections for 18 hours at 4 °C in a humidified chamber. Primary antibodies included: chicken-anti-GFP (1:500, Aves or Abcam), mouse anti-NeuN (1:200, Millipore), mouse anti-GFAP (1:500, NeuroMab, clone N206A/8 or Millipore, clone GA5), rabbit anti-SLC7A10, N-term (1:250–1:500, Acris, lot #FGI263), rabbit anti-beta-galactosidase (1:500, MP/Cappel), mouse anti-GLYT2 (1:500, Millipore), mouse anti-OLIG2 (1:500, Millipore), mouse anti-PSD95 (1:2000, NeuroMab), and mouse anti-GPHN (1:200, Synaptic Systems). Secondary detection was conducted at room temperature for 2 hours, using the following antibodies: goat anti-rabbit Alexa Fluor 488, 568, or 647, goat anti-mouse Alexa Fluor 488 or 594, goat anti-chicken Alexa Fluor 488, goat anti-guinea pig Alexa Fluor 594 (1:500, all from Invitrogen). Images were acquired using Zeiss Meta 510, Zeiss Axiovis, or Zeiss 800 and 880 Airyscan microscopes.

### Immunoblotting

Spinal cord homogenates were prepared in T-PER tissue protein extraction reagent (ThermoFisher Scientific) containing protease inhibitors (Roche). Protein concentration was measured by BCA assay (ThermoFisher Scientific). Protein equivalents from each sample were boiled in LDS (ThermoFisher Scientific) containing 2.5% β-mercaptoethanol and electrophoresed on 4–12% Bis-Tris acetate gels (BioRad). Protein was transferred to nitrocellulose (BioRad) in transfer buffer containing 25 mM Tris, pH 8.3, 192 mM glycine, 0.1% (w/v) SDS, and 20% methanol. Membranes were blocked for 1 h in 20 mM Tris, 500 mM NaCl, pH 7.5 (TBS) containing 5% milk, then incubated overnight at 4 °C with primary antibodies diluted in TBS-0.1% tween-20 (TBST) containing 5% milk. After washing in TBST, membranes were incubated with HRP-conjugated goat-anti-rabbit or goat-anti-mouse antibodies (GE Life Sciences) and detected using ECL reagents (GE Life Sciences). Primary antibodies included rabbit anti-beta-galactosidase (1:500, MP/Cappel), mouse anti-beta-tubulin (1:1000, Sigma), rabbit anti-SLC7A10, N-term (1:1000, Acris, lot #FGI263), mouse anti-GLYR (1:1000, Synaptic Systems), rabbit anti-VIAAT (1:1000, Aviva), rabbit anti-GLYT1 (1:1000, Aviva), and mouse anti-GLYT2 (1:1000, Millipore). Densitometry was performed using ImageJ.

### Amino acid quantification

Amino acid profiling was conducted at the West Coast Metabolomics Center at the University of California, Davis, using ALEX-CIS GCTOF (Automated Liner Exchange-Cold Injection System Gas Chromatography Time-of-Flight) mass spectrometry. Relative normalization was achieved as follows: for analyte *i* of sample *j,* analyte_ij_, _normalized_ = [(peak height of analyte_ij, raw_) / ∑ (peak heights of all annotated analytes_j_)] · [average peak height of all annotated analytes for all samples].

### Acute slice preparation

P10-P13 mice were deeply anesthetized with isofluorane. A ventral laminectomy was performed in ice-cold ACSF containing (in mM) NaCl 120, KCl 2.5, CaCl_2_ 2, MgCl_2_ 2, NaHCO_3_ 26, NaH_2_PO_4_ 1, glucose 11. Thoracolumbar segments were rested in an agar block fixed vertically on a Leica vibratome; 400 μm transverse (axial) slices were cut and then incubated in ACSF for 1 h at 32 °C before transferring to the recording chamber. ACSF used for both dissection and recording was saturated with 95% O_2_ and 5% CO_2_.

### Whole-cell patch-clamp recording

Whole-cell recordings were performed using an Axon 200B amplifier. To record and isolate mIPSCs, electrodes with a tip resistance of 3–5 MΩ were filled with a high-chloride internal solution containing (in mM) CsCl 147, Na_2_-phosphocreatine 5, HEPES 10, EGTA 1, MgATP 2, Na_2_GTP 0.3. Motor neurons were visualized by DIC optics using a 60x water-immersion objective. Cells were clamped at −70 mV and perfused with 1 μM TTX, 20 μM NBQX, and 50 μM AP5. To further isolate GABA-receptor-mediated or glycine receptor-mediated synaptic currents, 5 μM strychnine or 100 μM picrotoxin was perfused, respectively. To record and isolate AMPA/NMDA receptor-mediated excitatory synaptic currents (mEPSCs), electrodes were filled with an internal solution containing (in mM) Cs-methanesulfonate 115, CsCl 20, Na_2_-phosphocreatine 10, HEPES 10, EGTA 0.6, MgCl_2_ 2.5, MgATP 2, and Na_2_GTP 0.3; neurons were clamped at −70 mV and perfused with 1 μM TTX, 5 μM strychnine, and 100 μM picrotoxin. Electrophysiological studies were conducted by an investigator (Y.W.) masked to genotype with recordings from at least 10 motor neurons (n ≥ 3 biological replicates for each genotype).

### Statistical analysis

Quantitative experiments were conducted with n ≥ 3 age-matched biological replicates for each genotype, and littermates where possible. Cell counts were conducted using Imaris (Bitplane). Statistical analyses (one-way ANOVA with Bonferroni or Benjamini-Hochberg correction and Welch’s t-tests as appropriate) were conducted using Stata, version 12.1 (StataCorp, College Station, Texas) or R, version 3.1.3. mIPSCs were analyzed by Mini Analysis Program, version 6.0.3 (Synaptosoft, Georgia). α < 0.05 was considered statistically significant.

## Additional Information

**How to cite this article**: Ehmsen, J. T. *et al*. The astrocytic transporter SLC7A10 (Asc-1) mediates glycinergic inhibition of spinal cord motor neurons. *Sci. Rep.* **6**, 35592; doi: 10.1038/srep35592 (2016).

## Supplementary Material

Supplementary Information
