# Supplementary material for: The astrocytic transporter SLC7A10 (Asc-1) mediates glycinergic inhibition of spinal cord motor neurons
Source: Sci Rep. 2016 Oct 19;6:35592. doi: 10.1038/srep35592 (PMC5069678; doi:10.1038/srep35592)
Supplement: Supplementary Information [file srep35592-s1.pdf]

Supplementary Figure 1

The astrocytic transporter Slc7a10 (Asc-1) mediates glycinergic inhibition of spinal cord motor neurons

Jeffrey T. Ehmsen,[a,b,d] Yong Liu,[e] Yue Wang,[e] Nikhil Paladugu,[a] Anna E. Johnson,[a] Jeffrey D. Rothstein,[a,b,c] Sascha du Lac,[b] Mark P. Mattson,[b,e] Ahmet Höke[a,b,*]

[a]Department of Neurology,
[b]Solomon H. Snyder Department of Neuroscience,
[c]Brain Science Institute,
[d]Medical Scientist Training Program,
Johns Hopkins School of Medicine, Baltimore, Maryland, 21205, USA
[e]Laboratory of Neurosciences,
National Institute on Aging Intramural Research Program, Baltimore, Maryland, 21224, USA

*Corresponding Author:
Ahmet Höke, MD, PhD
Johns Hopkins School of Medicine
Department of Neurology
855 N. Wolfe Street, Rangos 248
Baltimore, MD 21205 USA
tel: 1-410-955-2227
email: ahoke@jhmi.edu

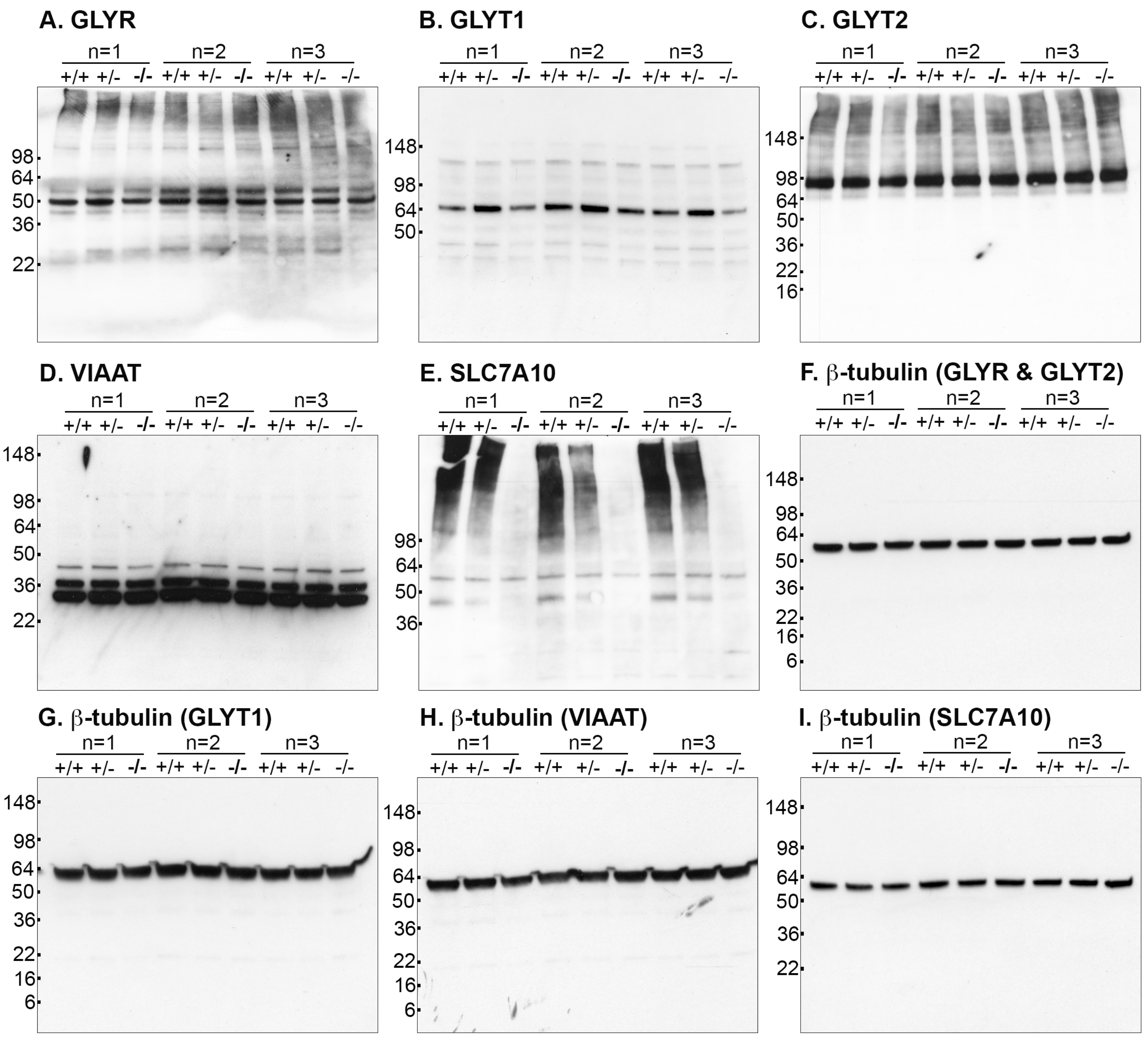
A. GLYR
B. GLYT1
C. GLYT2
D. VIAAT
E. SLC7A10
F. β-tubulin (GLYR & GLYT2)
G. β-tubulin (GLYT1)
H. β-tubulin (VIAAT)
I. β-tubulin (SLC7A10)
n=1
n=2
n=3
+/+ +/- -/- +/+ +/- -/- +/+ +/- -/-
148
98
64
50
36
22
16
6
